# Whither systems biology

**DOI:** 10.1098/rstb.2011.0074

**Published:** 2011-12-27

**Authors:** Anthony A. Hyman

**Affiliations:** Max Planck Institute for Molecular Cell Biology and Genetics, Pfotenhauer strasse 108, Dresden 01309, Germany

**Keywords:** systems biology, modelling, cell cycle

## Abstract

Cell biologists are interested in how complexity arises from the interaction of different molecules. However, cells are many orders of magnitude larger than the protein-binding interfaces. To bridge these vast difference in scales, biologists construct hierarchies of organization of cellular structures. I describe how systems biology provides an approach to bridge these different scales.

It has often been proclaimed that the future of cell cycle research is systems biology [1]. Why then is it that so many conversations at systems biology meetings revolve around the definition of systems biology? Defining the term has a direct impact on the funding, and therefore the immediate research goals, of biomedical research. Systems biology was driven by the realization that sequencing the genome and describing the individual parts, and understanding the chemistry of individual proteins did not explain how biological systems worked. Perceptions of systems biology fall between one of two extremes—the modellers and the collectors: both ultimately want to explain biological outputs (gene expression or development as examples) as the result of biological inputs (transcription factor phosphorylation or growth factor expression). At one extreme is the collector who relies on a deterministic view of the world and has a complete list of parts and interactions (the top-down approach). Collection has been propelled by an explosion in genomics, bioinformatics, laboratory automation and imaging techniques. At the other extreme are modellers, who believe biological systems should be approached from physical chemistry principles (the bottom-up approach). Since these two approaches would seem complementary at first glance, one might wonder why people of these two communities tend to lock horns. As the field grew, funding administrators were often left scratching their heads as to what to fund and how. The struggle to define systems biology comes with large rewards, as the winners will collect large funds from national and transnational funding efforts. For instance, in Germany, the systems biology of liver programme has been heavily supported by the BMBF (Bundesministerium fur Bildung und Forschung; a government funding organization). Those who work on liver have lived high off the hog, while those who work on kidney (for example) have had to scrape along with the masses.

Barriers to defining systems biology come from, in part, the perceptions of scale in biological analysis [2]. To understand their problems, biologists have to work across vast scales of time and space. Organisms can be a billion times larger than the protein-binding interfaces critical for their development. Similarly, it might take years for an organism to reach sexual maturity, but the chemical transformations or conformational changes in proteins orchestrating the process require microseconds. To organize up to nine orders of magnitude, a biologist instinctively constructs a (one-within-another) hierarchy within which to organize their research. Indeed, this idea was first enunciated by Hebert Simon in 1962 [3], who proposed that complex systems are composed of subsystems [4]. As an example, figure 1 shows the scales of organization behind the cell biology of tissue organization. To understand tissues, we have to understand the collective behaviour of cells. To understand cells, we need to understand the collective behaviour of organelles. And thus, we continue downwards in scale until we reach the individual functional groups of amino acids in proteins or nucleic acids. For example, when understanding how microtubules grow, we need to understand the collective behaviour of tubulin molecules. It is not really necessary to understand how tubulin works to understand how a cell behaves. But we do need to understand tubulin function to understand microtubule function. At each level, understanding comes from interplay between the closest levels of organization. We understand the organization of tissues by studying the behaviour of cells. But we also understand the behaviour of cells by studying the tissues of which they form a part. We understand the organization of microtubules by studying the properties of tubulin, but we understand the properties of tubulin by studying microtubules. Physics has generally worked harmoniously within these levels of hierarchy. Fluctuations in macromolecular space do not require an understanding of quarks. However, biologists have always struggled with the levels of hierarchy. Do those studying tissues (developmental biologists) need to understand protein structures?

**Figure 1.**
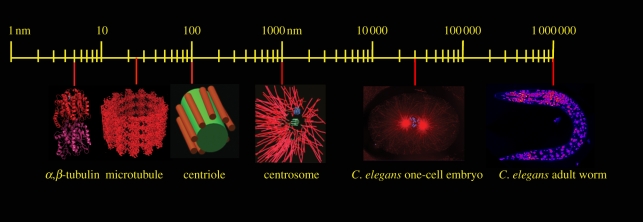
Scale in biological analysis. The figure illustrates components of a *Caenorhabditis elegans* mitotic spindle at different length scales, spanning about six orders of magnitude.

Models provide a key tool to bridge these different levels of complexity [4]. Models are formal representations of the system at one level of organization. The new (sometimes called ‘emerging’) properties of a model that are not already part of its definition, represent phenomena at the next level of organization (for an excellent description of these ideas see [5]). Some examples are diffusion emerging from a microscopic description of interacting molecules in a fluid; properties of gene networks based on rules for gene interactions; spindle oscillations emerging from the interaction of many cortical motors and microtubules; behaviours of signalling cascades (vision, olfaction) based on molecular interactions of ion channels, G proteins, etc.; and behaviour of a neural network based on interactions of individual neurons.

The term ‘modelling’ describes the procedure to construct a model of a system, which can mimic observed behaviours. If models are formulated in a mathematical language and describe values of quantities that can be observed experimentally, they can be simulated on computers and their behaviours can be compared with quantitative experiments. A key problem with modelling is that one requires a conceptual basis and a systematic strategy to be able to construct useful and insightful models. Without a systematic approach, models are often meaningless. In many cases, models generate properties that are similar to a real system by accident. Any complicated model can be made to reproduce data just by adding additional features. For example, if the number of parameters (such as kinetic constants) of a model is large, a vast variety of behaviours can be generated and thus a comparison with experimental data can have high similarity but be of little significance. A key goal of modelling is to discover how rather simple models can be constructed that generate behaviours of systems because key mechanisms are captured correctly. If this can be demonstrated clearly for a given system, real insight is gained and there is a good chance that such a model can make predictions for new experimental conditions. Two things are key to the success of modelling. The first is quantitive data collection. Not the one plus, two plus or three plus of the cloning generation, but numbers and confidences. The second is a theoretical framework of concepts and methods that lays the basis to build models, guided by an increasing amount of experimental information. Therefore, one way to describe systems biology is the following: it is the approach of collecting quantitative biological information at one level of complexity, and using it to build models that describe the next level of complexity.

This view of systems biology explains why it is so hard to describe a systems biology department, or a systems biology meeting. This is because systems biology is an approach and not a field. Traditional fields that link different levels of organization already exist. To give a few examples, developmental biology tries to understand the development of tissues by looking at cells. Cell biologists try to understand the organization of cells by looking at protein complexes and organelles; structural biologists try and understand the organization of proteins by looking at three-dimensional organization of atoms. Importantly, completely different strategies of data collection and modelling will be needed for taking systems approaches in each one of these fields. Modelling strategies for cellular behaviour are unlikely to be useful for modelling protein structure. Even within one field, different strategies will be necessary. Consider for example, a cellular structure such as the mitotic spindle. At different scales, different concepts become relevant. For example, a description of microtubules on the molecular scale has to consider the tubulin lattice as well as GTP and GDP states of monomers [6]. The same structure described on a larger scale is governed by polymer physics such as thermal bending modes and buckling forces [7]. A description of a mitotic spindle thus requires an interrelated set of models at different scales capturing different aspects such as filament dynamics, chromatin condensation, molecular diffusion, kinetochore activation, cell cycle signalling, etc. In this regard, systems biology is similar to an approach such as imaging, which by analogy would be called imaging biology. Different forms of imaging are required to look at proteins (X-ray diffraction), protein complexes (electron tomography), protein dynamics (atomic force microscopy), protein localization (stimulated emission depletion) or cellular behaviour (confocal microscopy). In the same way that imaging approaches are now embedded in all fields of biology (there are few meetings on imaging, and fewer departments), so systems approaches need to be an integral part of all biological analysis, and should be integrated into existing departments. It is not enough for physicists to come into biology and bring their quantitative and analytical skills. Quantitative skills must be a fundamental part of the training of all biologists. No longer can biology be the refuge of those (like myself) who were no good at mathematics.

A related issue is that quantitative data collection will be essential at all levels of analysis of biological systems. This applies not only to the collection of data for small-scale models that I have described above, but also for large-scale ‘omics’-based experiments that seek to catalogue and analyse systems by systematic perturbation of genes. The analysis of such screens is and will be valuable for systems analysis if the data collected is quantitative. For instance, genome-wide screens for genes required in the cell cycle will be much more valuable if they assess such issues as levels of kinase activity, or levels of proteolysis rather than gross phenotypic analysis. Equally powerful is to study how gene networks change after perturbation, rather than individual genes. The ultimate goal will be to test small-scale models using large-scale genetic screens. Again, these analyses will involve a component of modelling. It is difficult to make sense of this huge amount of data without informatics approaches that help to catalogue the data, and to model the expected outcomes of such experiments.

Given that theory will be essential to understand biology, why is it that theory has become less important in biology over the last 30 years? Why is the study of the cell cycle still dominated by collection of parts and mapping of phosphorylation sites? There are two reasons for this. The first is that theoretical biology was somewhat discredited precisely because it was too hard to experimentally verify and because some early attempts were generally based on insufficient data. Not enough was known to constrain the theory, and the experimental techniques were often too primitive. Where experimental techniques were strong, such as in the peripheral nervous system, there has always been a strong component of theory. Therefore, with the molecular biology revolution, biologists settled down to describe again. Not as in the nineteenth century, where the key was to describe the species. This time the goal was to describe the genes and their relationship with the phenotype of the cell of organism. The study of an individual protein or protein complex and its general role in the biology of an organism did not need a theory. Theory is becoming important again, because the complexity of the problems that we are interested in is moving beyond single proteins or complexes, and into the problem of systems. Certainly, the cell cycle itself can only be understood as a system, especially when the upstream and downstream responses are factored in. In exactly the same way, theories of ecology and evolution were necessary to make sense of all the work of the naturalists, as they collected and documented species. Understanding the organization of biological systems such as the cell cycle in the molecular biology era will require a strong component of theory which will be necessary to build the models and design the experiments necessary to test them.
